# Ultrastructure Organization of Human Trabeculae Assessed by 3D sSAXS and Relation to Bone Microarchitecture

**DOI:** 10.1371/journal.pone.0159838

**Published:** 2016-08-22

**Authors:** Marios Georgiadis, Manuel Guizar-Sicairos, Oliver Gschwend, Peter Hangartner, Oliver Bunk, Ralph Müller, Philipp Schneider

**Affiliations:** 1 Institute for Biomechanics, ETH Zurich, Zurich, Switzerland; 2 Paul Scherrer Institut (PSI), Villigen, Switzerland; 3 Bioengineering Science Research Group, Faculty of Engineering and the Environment, University of Southampton, Southampton, United Kingdom; Pennsylvania State Hershey College of Medicine, UNITED STATES

## Abstract

Although the organization of bone ultrastructure, i.e. the orientation and arrangement of the mineralized collagen fibrils, has been in the focus of research for many years for cortical bone, and many models on the osteonal arrangement have been proposed, limited attention has been paid to trabecular bone ultrastructure. This is surprising because trabeculae play a crucial role for the mechanical strength of several bone sites, including the vertebrae and the femoral head. On this account, we first validated a recently developed method (3D sSAXS or 3D scanning small-angle X-ray scattering) for investigating bone ultrastructure in a quantitative and spatially resolved way, using conventional linearly polarized light microscopy as a gold standard. While both methods are used to analyze thin tissue sections, in contrast to polarized light microscopy, 3D sSAXS has the important advantage that it provides 3D information on the orientation and arrangement of bone ultrastructure. In this first study of its kind, we used 3D sSAXS to investigate the ultrastructural organization of 22 vertebral trabeculae of different alignment, types and sizes, obtained from 4 subjects of different ages. Maps of ultrastructure orientation and arrangement of the trabeculae were retrieved by stacking information from consecutive 20-μm-thick bone sections. The organization of the ultrastructure was analyzed in relation to trabecular microarchitecture obtained from computed tomography and to relevant parameters such as distance to trabecular surface, local curvature or local bone mineralization. We found that (i) ultrastructure organization is similar for all investigated trabeculae independent of their particular characteristics, (ii) bone ultrastructure exhibiting a high degree of orientation was arranged in domains, (iii) highly oriented ultrastructural areas were located closer to the bone surface, (iv) the ultrastructure of the human trabecular bone specimens followed the microarchitecture, being oriented mostly parallel to bone surface, and (v) local surface curvature seems to have an effect on the ultrastructure organization. Further studies that investigate bone ultrastructure orientation and arrangement are needed in order to understand its organization and consequently its relation to bone biology and mechanics.

## 1 Introduction

Bone’s remarkable mechanical properties are a consequence of its hierarchical structure. At the basis of this structure lies the mineralized collagen fibril, bone’s ultrastructural unit [1,2]. The organization of the ultrastructure has been shown to be important for the strength of bone tissue and for other tissues and materials, affecting the mechanical properties at the apparent level [3–6] as well as at the microscale [7–10]. Various techniques have been used to assess the organization of bone’s ultrastructure, namely the orientation and arrangement of mineralized collagen fibrils. The most common techniques for assessing samples with a field of view at the millimeter or centimeter scale are linearly and circularly polarized light microscopy (PLM) [11,12], second harmonic generation (SHG) imaging [13,14], polarized Raman imaging and Fourier transform infrared (FTIR) imaging [15,16], and small-angle and wide-angle X-ray scattering (SAXS and WAXS) [17–20], of which only small-angle scattering tensor tomography [19] and six-dimensional SAXS tomography [20] can provide the information in a tomographic, non-destructive way. For samples at the micrometer scale, phase nanotomography [21–23], ptychography [24,25] and volume electron microscopy [26,27] have demonstrated their capabilities in providing invaluable information concerning fibril organization in bone, the former two being tomographic, i.e. non-destructive, techniques. A comprehensive review of the methods used for studying bone ultrastructure organization, with their advantages and disadvantages, is given in [28]. A novel technique based on scanning SAXS (sSAXS), called 3D sSAXS, which we developed recently [29], allows deriving the local 3D orientation and arrangement of the ultrastructure of millimeter-sized samples in a spatially resolved way. The technique works for thin sections (prepared with the use of a microtome), however, if applied to consecutive sample sections, it provides the 3D ultrastructure orientation of a 3D volume, and can thus be used to reconstruct ultrastructure organization maps within bulk samples.

The organization of the bone ultrastructure has been the topic of extensive study in cortical bone, where many models have been proposed for the osteon, such as the rotated [30] and the twisted plywood model [31] based on electron microscopy observations, the helicoidal plywood model [32] based on X-ray diffraction data, or the oscillating plywood model [22], which is the only model that is derived from (direct) 3D observations using phase nano-tomography [22]. However, the study of the ultrastructure organization in trabecular bone has been given limited attention up to now, with few studies published only [27,29,33]. This is rather unexpected, since trabecular bone plays a crucial role in the mechanical strength of bone sites such as the vertebrae and the femoral head. In addition, the higher metabolic rate of trabecular bone compared to cortical bone [34] makes it particularly vulnerable to pathological bone conditions such as osteoporosis, which is the most common metabolic bone disease. The scarcity of studies on trabecular ultrastructure organization is probably due to the fact that trabecular bone does not have a repetitive unit such as the osteon, and due to the greater variability in trabecular architecture vs. cortical bone structure, when compared at the same anatomical site between subjects and within the same subject followed over time. And–to add to the complexity of the issue at hand–for the same subject, site and point in time, one can find trabeculae of different alignment, type (rods or plates), size and orientation, and regions of different remodeling age inside trabeculae. Therefore, in the best case, a systematic study of trabecular bone ultrastructure should include trabeculae of different alignment, types (rods or plates), from different anatomical sites, and from different subjects at varying ages.

To demonstrate the suitability of the 3D scanning SAXS method (referred to as “3D sSAXS”) for studying bone ultrastructure, we examined ultrastructure orientation and arrangement derived by 3D sSAXS, when correlated with two-dimensional (2D) results from commonly employed polarized light microscopy (PLM). Furthermore, we present the first investigation of the organization of the ultrastructure in human vertebral trabecular bone for 22 trabeculae of different alignment, types and sizes, and from subjects at different decades during their lifetime.

## 2 Materials and Methods

### 2.1 Sample preparation

**Fig 1:**
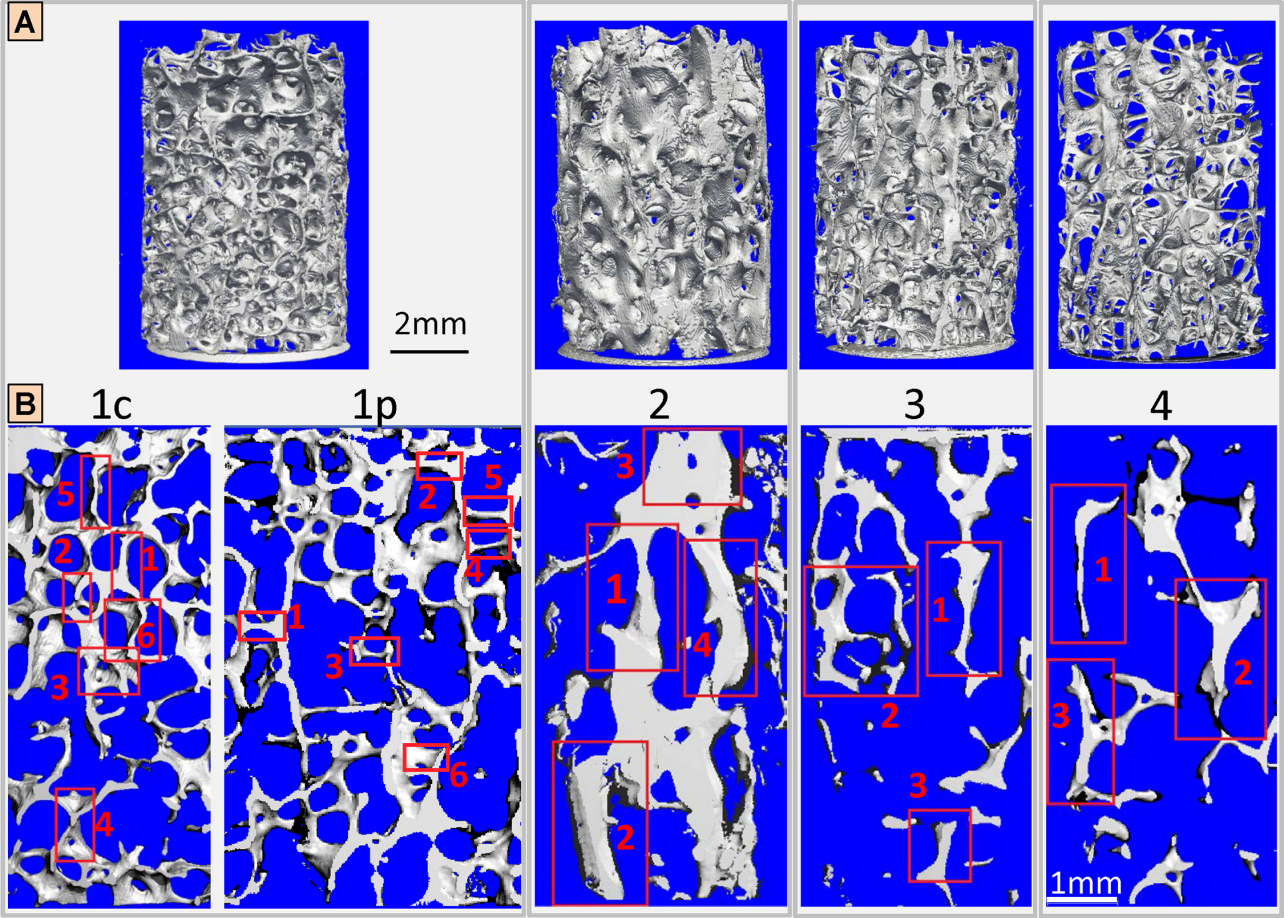
Vertebral specimens of the 4 subjects and trabeculae that have been studied. (A) Cylindrical vertebral cores from 4 different subjects (see Table 1), imaged using synchrotron radiation-based computed tomography (SR CT) at 7.4 μm voxel size. The symmetry axis of the cylinder corresponds to the craniocaudal direction. Differences in bone mass are apparent, corresponding to different ages of the donors (see Table 1). (B) The bone samples selected from the cylindrical cores consisted in sequences of 20-μm-thick histological sections, including the selected regions of interest (red frames), which designate trabeculae analyzed by 3D scanning small-angle X-ray scanning (3D sSAXS). Sample 1c mostly contained trabeculae with their long axis in the craniocaudal direction, while Sample 1p had preferentially trabeculae at a perpendicular direction. Selected trabeculae from Sample 1c were mostly plate-like because this was by far the most common trabecular type found for the craniocaudal direction. On the other hand, perpendicular trabeculae for Sample 1p were mostly rod-like, whereas selected trabeculae from the other subjects (2–4) exhibited more irregular shapes, in-between plates and rods.

Cylindrical cores (6 mm diameter, 9 mm height) (Fig 1) were extracted from the trabecular compartment of vertebrae from 4 subjects in a craniocaudal direction using a hollow diamond drill. Information on the subjects and the cylindrical cores can be found in Table 1. The vertebral specimens used in this study were kindly provided by Dr. Werner Schmölz, Department for Trauma Surgery, Innsbruck Medical University, Innsbruck, Austria, and have also been used in previous studies [19,29,35]. The human tissue was obtained from the Department for Anatomy, Histology, and Embryology at the Innsbruck Medical University with the written consent of the donor and following the anonymous analysis scheme, whereby the donors were anonymized during the tissue collection process. Hence no further ethical approval was required under Austrian law, as explicitly stated in the guidelines of the Ethical Review Committee, ERC, Innsbruck Medical University (decision at meeting no. 274 on 19 February 2009 regarding research project review through the ERC, paragraph B). All procedures thereafter were in accordance to Swiss law, the Guideline on Biobanking of the Swiss Academy of the Medical Sciences (2006) and the Swiss ordinance 814.912 on the contained use of organisms.

**Table 1:** Summary of subjects, bone samples, and histological sections used in the study.

	Subject 1	Subject 2	Subject 3	Subject 4
**Subject gender**	Female	Male	Male	Male
**Subject age**	50	27	52	77
**Vertebra**	Th12	Th12	Th10-11*	Th10-11*
**BV/TV (%)**	12.8	21.8	10.3	4.8
**SMI**	1.13	1.35	1.42	1.47
**DA**	1.20	1.42	1.47	1.46
**Tb.Th (mm)**	0.08	0.15	0.09	0.06
**Tb.Sp (mm)**	0.52	0.55	0.79	1.28
**Nr of trabeculae**	6 craniocaudal (**1c**)	4	3	3
	6 perpendicular (**1p**)			
**Nr of sections**	24 craniocaudal (**1c**)	10	10	10
	20 perpendicular (**1p**)			

* exact thoracic number unknown

Morphometric measures (BV/TV, SMI, DA, Tb.Th, Tb.Sp) [36] derived from lab-based micro-computed tomography scans at a voxel size of 12 μm [35]. Abbreviations: BV/TV, (trabecular) bone volume fraction; SMI, structure model index; DA, degree of anisotropy; Tb.Th, trabecular thickness; Tb.Sp, trabecular separation

Quantitative morphometric measures of the trabecular network (Table 1) have been derived from lab-based micro-computed tomography scans (μCT 40; Scanco Medical AG, Brüttisellen, Switzerland) at a voxel size of 12 μm, an electrical potential across the X-ray tube of 70 kVp, and an X-ray tube current of 114 μA [35], using proprietary software (Image Processing Language or IPL; Scanco Medical AG, Brüttisellen, Switzerland) and following present guidelines for the assessment of bone microstructure in rodents using micro-computed tomography [36]. The samples were consequently cleaned from soft tissue and imaged in saline using synchrotron radiation-based computed tomography (SR CT) at the TOMCAT beamline of the Swiss Light Source (SLS) at the Paul Scherrer Institut (PSI), Villigen, Switzerland. A 7.4 μm voxel size and a beam energy of 17.5 keV were selected and 1081 projections were acquired per sample [35].

Subsequently, the samples were dehydrated through a series of ethanol baths and embedded in polymethyl methacrylate (PMMA), before a microtome (HM 355S; Thermo Fisher Scientific Inc., USA) was used to cut them into consecutive 20 μm-thick tissue slices, which were directly attached to a 12 μm-thick Kapton adhesive tape (T2000023; Benetec, Wettswil, Switzerland). In order to investigate different trabecular alignments, two samples of the first cylindrical core from Subject 1 (Table 1) were prepared: the first sample comprised six trabeculae with their long axis in the craniocaudal direction (Sample 1c), whereas the second sample contained six trabeculae with their long axis perpendicular to the craniocaudal direction (Sample 1p). Due to cutting artifacts that occur in trabecular structures orthogonal to the cutting direction, the bone core had to be rotated by 90° for sectioning the perpendicular trabeculae of the second sample, to make sure that their long axis was aligned along the cutting direction. Regions of interest (ROIs) that included trabeculae spanning over consecutive histological sections were selected under a microscope. Subsequently, the sample sections were mounted on custom-made aluminum holders for the 3D sSAXS experiments (Fig 1 in [29]).

Since excision and until the experimental procedure described in this manuscript, the vertebral specimens were stored at a temperature of -80°C for a period of approximately 8 years. To the best knowledge of the authors, there is no known effect of the different sample preparation steps adopted here on the organization of bone ultrastructure, which is thus considered to reflect physiological conditions.

### 2.2 3D scanning small-angle X-ray scattering (3D sSAXS)

**Fig 2:**
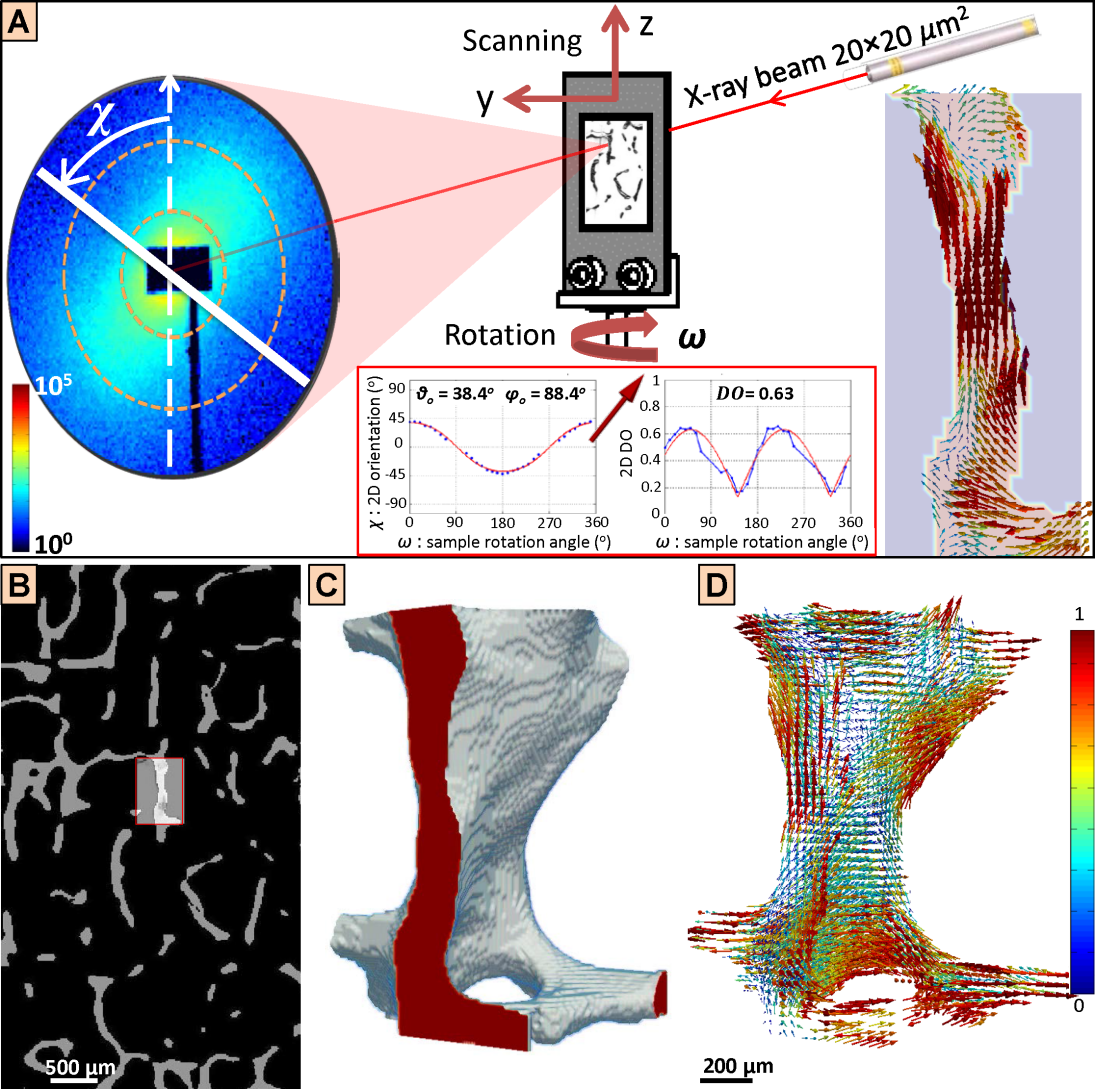
3D scanning small-angle X-ray scattering (3D sSAXS) experimental procedure, providing spatially resolved and quantitative 3D ultrastructure information for trabecular bone. (A) The histological bone section was mounted on a metal holder, and raster-scanned with a micro-focus X-ray beam, at a step size of 20 μm, for 10 rotation angles *ω*. The 2D orientation angle *χ* as well as the 2D degree of orientation (DO) were extracted from each diffraction pattern [38], for a *q*-range of 37.9–75.8 μm^−1^ (orange dashed circles), suitable to retrieve the orientation of mineralized collagen fibrils in the bone tissue. The 3D information (polar and azimuthal angles *θ*_ο_ and *φ*_ο_ and the 3D DO) was derived by fitting the experimental data to sinusoidal equations (red inset), as described in [29]. For further experimental details, please consult [29]. The result for all points in the region of interest (ROI) is the orientation map shown at the right, where the ultrastructure arrangement of each 20 × 20 × 20 μm^3^ bone volume is represented by a vector, whose direction represents the main orientation of the ultrastructure and the length and color stand for the DO. (B) The histological bone section of the investigated ROI is registered to the SR CT-derived volume, to identify the corresponding digital section in the CT volume (gray bone on black background). The X-ray transmission information recorded by a photodiode on site during the 3D sSAXS experiments (red inset) is registered to the digital SR CT section, which allows identifying every point of the ROI within the 3D bone structure, and thereby spatial mapping between ultrastructural information obtained from 3D sSAXS and microstructural information retrieved from SR CT. C) 3D bone structure from SR CT, which corresponds to all the points within the pre-selected ROIs that have been analyzed using 3D sSAXS. A virtual cut (indicated as red surface) has been performed to the structure at the position of the ROI in (A). D) The 3D sSAXS information of the whole trabecula can be plotted as a 3D map that represents the ultrastructure orientation, where the direction of the vectors corresponds to the ultrastructure orientation and the vector length and color indicate the DO in a quantitative fashion (given by the colormap on the right). A virtual cut similar to that in (C) has been performed at the position of the 2D orientation map at the right part of (A).

The 3D sSAXS experiments were performed at the coherent small-angle X-ray scattering (cSAXS) beamline of the Swiss Light Source (SLS) at the Paul Scherrer Institut (PSI), Villigen, Switzerland, according to the procedures described in [29] (Fig 2). Briefly, a monochromatic beam at 12.4 keV and of 20×20 μm^2^ (FWHM) spot size was used to raster-scan the selected ROIs (Fig 1). The X-ray diffraction patterns were collected using a PILATUS 2M detector (sample-to-detector distance = 7.150 m) with 50 ms exposure time and 3 ms readout time, while the fast-axis motor along the *y*-axis (Fig 2) moved continuously. The samples were raster-scanned at 10 different angular positions, ±30° and ±60° around 0° and 180°, with a scanning time of ~5 min for each angular step and for a 1.0×1.5 mm^2^ ROI. The “reference” orientation or 0° angle corresponded to the configuration where the bone section was aligned perpendicular to the incoming X-ray beam (Fig 2). The scan step was adjusted to 20·cos(ω) μm for each rotation angle ω, so that in every step the X-ray beam would probe a bone volume approximately equal to 20 × 20 × 20 μm^3^. The X-ray transmission of the sample was measured by a silicon photodiode mounted on the beam stop. The transmission images were registered rigidly [37] to the 0° reference image, so that the same 20 × 20 × 20 μm^3^ bone volume could be identified for all angular steps.

The 2D orientation angle (*χ*) and the projected degree of orientation (2D DO) were extracted from each diffraction pattern according to the analysis given in [38] for a *q*-range of 37.9–75.8 μm^−1^, delineated by dotted lines in Fig 2A. In order to retrieve the corresponding 3D quantities, namely the ultrastructure orientation, defined by the spherical coordinate angles *φ*_*ο*_ and *θ*_*ο*_, and the 3D degree of orientation (3D DO), the experimental data for each 20 × 20 × 20 μm^3^ bone volume (2D orientation angle *χ*, sample rotation angle *ω* and 2D DO) were fitted to the equations:

$$\tan\left(\chi \right)=\frac{sin(\theta_{o})\sin\left(\varphi_{o}+\omega \right)}{cos(\theta_{o})}=tan(\theta_{o})\sin\left(\varphi_{o}+\omega \right).$$ Eq (1)

$$(2D\;DO)=(3D\;DO)\sqrt{\sin^{2}\theta_{ο}∙\sin^{2}\left(\varphi_{ο}+\omega \right)+\cos^{2}\theta_{ο}}.$$ Eq (2)

A comprehensive analysis of the involved parameters, the equations and their derivation is provided in [29]. Examples of data fitted to the equations are shown in the red inset of Fig 2A. The above analysis resulted in 2D maps of the 3D organization of the mineralized collagen fibrils (Fig 2A, right side). For the reconstruction of 3D orientation maps of whole trabeculae, the 2D maps retrieved from 3D sSAXS had to be registered to the 3D bone structure. The registration procedure was as follows: the microscope image of every 20 μm-thick histological bone section, wherein the different ROIs have been pre-selected (Fig 1), was registered by employing a 2D-to-3D registration algorithm [39] to the 3D bone structure derived from initial SR CT scans that were performed prior to physical sectioning. As a result, each raster-scanned ROI could be identified in a virtual SR CT section. The exact position of the ROI in the virtual section, and thus in the 3D bone structure, was retrieved by calculating the cross-correlation of the 2D transmission information (provided on site during the 3D sSAXS experiments by the silicon photodiode) with the registered virtual SR CT grayscale section of the 3D bone structure, using Matlab (2014a; The Mathworks, Natick, MA, USA). An example for this merging or spatial mapping process between ultrastructural information obtained from 3D sSAXS and microstructural information retrieved from SR CT in pre-defined ROIs is shown in Fig 2B.

### 2.3 Polarized light microscopy

**Fig 3:**
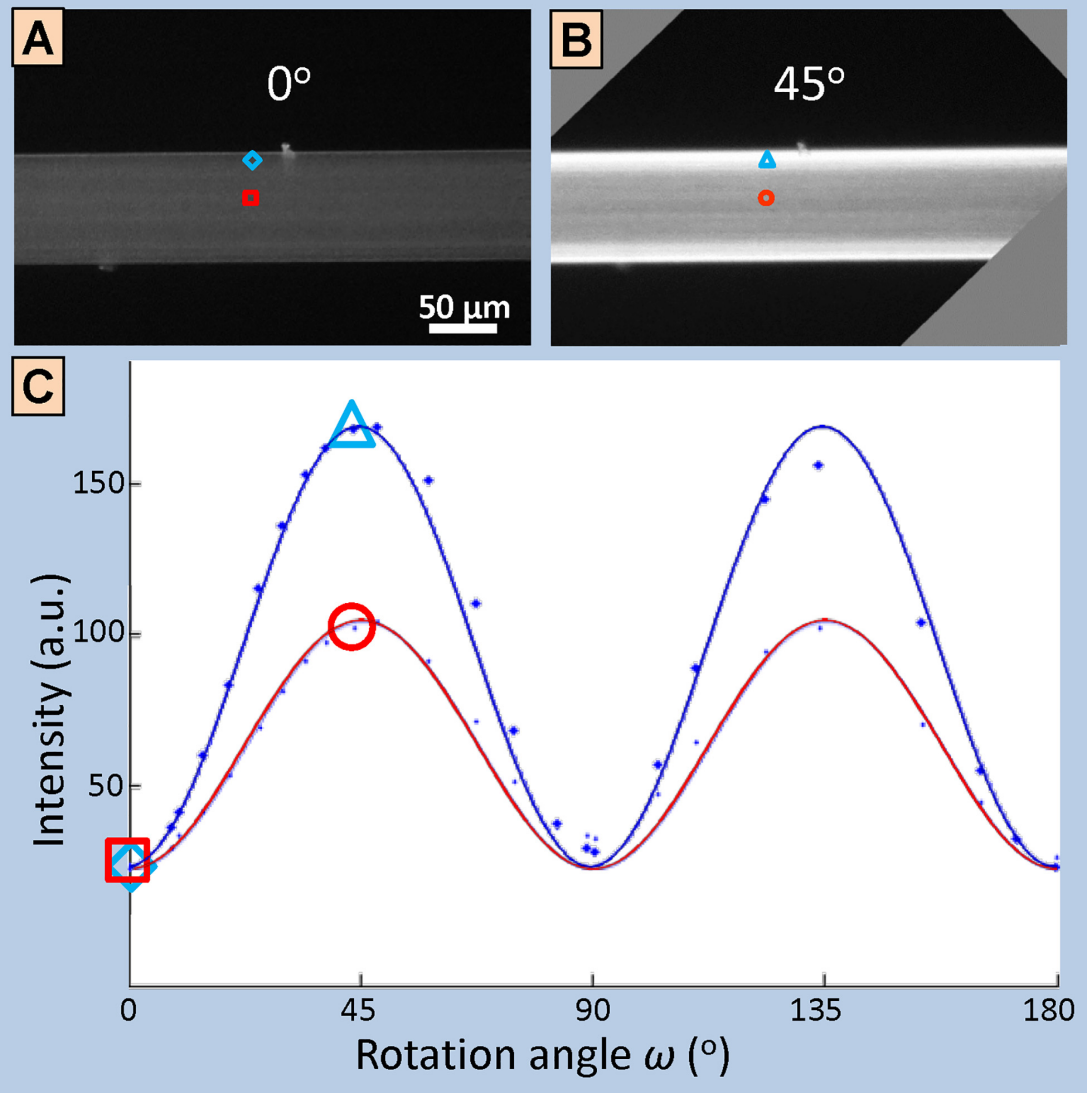
Procedure to retrieve ultrastructure orientation using linearly polarized light microscopy. (A-B) Linearly polarized light microscopy (PLM) images of a polymer fiber with ultrastructure parallel to the longitudinal axis, under two different rotation angles: (A) at 0°, where the ultrastructure is aligned parallel to the polarizer direction (extinction angle) and (B) at 45° from both the polarizer and the analyzer, where the recorded intensity is at its maximum for all points of the fiber. (C) Plot of the PLM intensities of the fiber points in the red and blue boxes shown in (A-B). Intensity is fitted to a sinusoidal curve (see S1 File for more details), where the phase shift reveals the in-plane orientation angle of the ultrastructure (~0° for this case, because the fiber is horizontally oriented at 0°) and where the amplitude is a measure of the in-plane degree of orientation and/or the thickness of the object. Here, the amplitude is mainly a measure of the local sample thickness, while for the case of the 20 μm-thick bone sections the amplitude is a measure of the local in-plane degree of orientation.

Polarized light microscopy (PLM) has been widely used to study bone ultrastructure organization in 2D, by exploiting the (positive) intrinsic birefringence of collagen [40,41]. Circular polarized light microscopy is the most commonly used imaging modality of PLM to investigate the orientation and arrangement of bone ultrastructure, mainly because of the concentric rings that are formed by the lamellae of an osteon [42], without the “Maltese cross” effect that linearly polarized light entails [11] (see Fig 1 in [11] for instance, and the S1 File in this publication for a mathematical explanation). However, the intensity of a homogeneously thick bone section in circular PLM depends on i) the out-of-plane angle of the collagen fibers, ii) the mineralization level of the tissue (alternatively the collagen density), and iii) the degree of orientation (DO) of the ultrastructure at this point. Thus, it is not possible to quantitatively derive the out-of-plane fibril orientation using circular PLM. On the other hand, by using linear PLM one can derive the orientation of the collagen fibrils in the plane of the investigated sample section, perpendicular to the light path. In linear PLM, the intensity depends on the angle between the laser polarization direction and the in-plane orientation of the collagen fibrils, which allows deriving the in-plane orientation of the fibrils by rotating either the sample or the polarization of the light source, and by fitting the recorded intensity to a sinusoidal curve [12] (see Fig 3 and the S1 File for a mathematical explanation). The angular offset of the curve provides the fibril in-plane orientation angle, the amplitude range of the curve is a measure of the degree of orientation (anisotropy level), and the intensity offset indicates the collagen density and out-of-plane angle orientation.

To validate 3D sSAXS for studying bone ultrastructure, we correlated 3D sSAXS results with 2D outcomes from more conventional (2D) PLM. To this end, linear PLM data were collected on a polarized light microscope (Axioskop2 MOT; Zeiss, Germany), with a 5X objective (NA = 0.12, Achrostigmat; Zeiss, Germany) that resulted in a pixel size of 1.3 × 1.3 μm^2^ and a field of view of approximately 1290 × 880 μm^2^. The images have been acquired with a color chilled 3CCD camera and an appertaining controller (C5810; Hamamatsu, Japan). Light intensity was adjusted so that the maximum pixel intensity did not exceed the dynamical range of the detector. Source stability was ensured through the use of an optical power meter (Nova II; Ophir, Israel) and the acquired images were flat field-corrected to compensate for the uneven distribution of the incident light. Analysis of image reproducibility (data not shown here) in terms of recorded intensities indicated that higher light intensities provided most reproducible results.

Bone samples were rotated manually in steps of 4–7 degrees over 180°, leading to sets of 26–45 images for every sample. In each step, a polarized and a bright-field image were acquired. The exact rotation angle was precisely quantified later, by registering each bright-field image to the one acquired at 0°, by using a 2D image registration algorithm [39]. Image registration also compensated for small translations caused by the manual rotation of the stage. After flat-field correction, polarized images were converted into 8-bit grayscale images and filtered with a Gaussian filter (3×3 kernel size) using Matlab. Images were scaled and interpolated from the original pixel size of 1.3 × 1.3 μm^2^ to 20 × 20 μm^2^, to match the pixel size of the 3D sSAXS experiments. For both the rotation as well as the scaling and interpolation, which were performed in one single step, a computationally inexpensive nearest neighbor scheme was preferred over three other alternatives (linear, cubic, and spline interpolation), after a quantitative analysis provided similar results for all interpolation schemes (data not shown here).

### 2.4 Validation of 3D sSAXS

For validation of 3D sSAXS in terms of assessment of the ultrastructure orientation, the same samples have been assessed and evaluated using (2D) linear PLM, which was considered as gold standard in this study. Subsequent correlation between the ultrastructure orientation retrieved from 3D sSAXS and PLM provided quantitative measures for the actual validation of 3D sSAXS.

Four circular ROIs within trabecular bone tissue from four different sections of the trabecular core of subject 2 were assessed with both 3D sSAXS and linear PLM. ROIs were circular to accommodate for the sample rotation involved in linear PLM as described earlier (cf. Fig 3). Bright-field PLM images at 0° were registered with 3D sSAXS data, using a 2D image registration algorithm [39]. One has to bear in mind that linear PLM provides only the in-plane orientation of the fibrils. Thus, for the quantities to be comparable, the 3D organization information derived from 3D sSAXS has to be reduced to its 2D components. This was performed by projecting the 3D orientation vector from 3D sSAXS, which represents the 3D orientation of the ultrastructure in a specific 20 × 20 × 20 μm^3^ bone volume, to the plane of the histological section.

In order to quantitatively compare the two orientation vectors from the two different imaging techniques, the vectorial information was broken up into the angle and the length of the vector. These two parameters were compared separately. Since linear PLM has the inherent ambiguity of ±90° for the in-plane orientation, the angle closer to the 3D sSAXS angle was selected (see S1 File). Since the degree of orientation (DO) is measured using distinct physical phenomena and in a different way for each technique–the range of intensity for linear PLM [12] versus the maximum value of the fit in Eq 2 for 3D sSAXS–the histograms for the DO had to be equalized between results of the two techniques. For that reason, the mean DO and its standard deviation has been set to 0.5 and 0.25, respectively, where DO values further apart from the mean than two standard deviations were accumulated at a distance of two standard deviations from the mean value, so that normalization has not been influenced by extreme values.

### 2.5 Analysis of bone ultrastructure and trabecular microarchitecture

**Fig 4:**
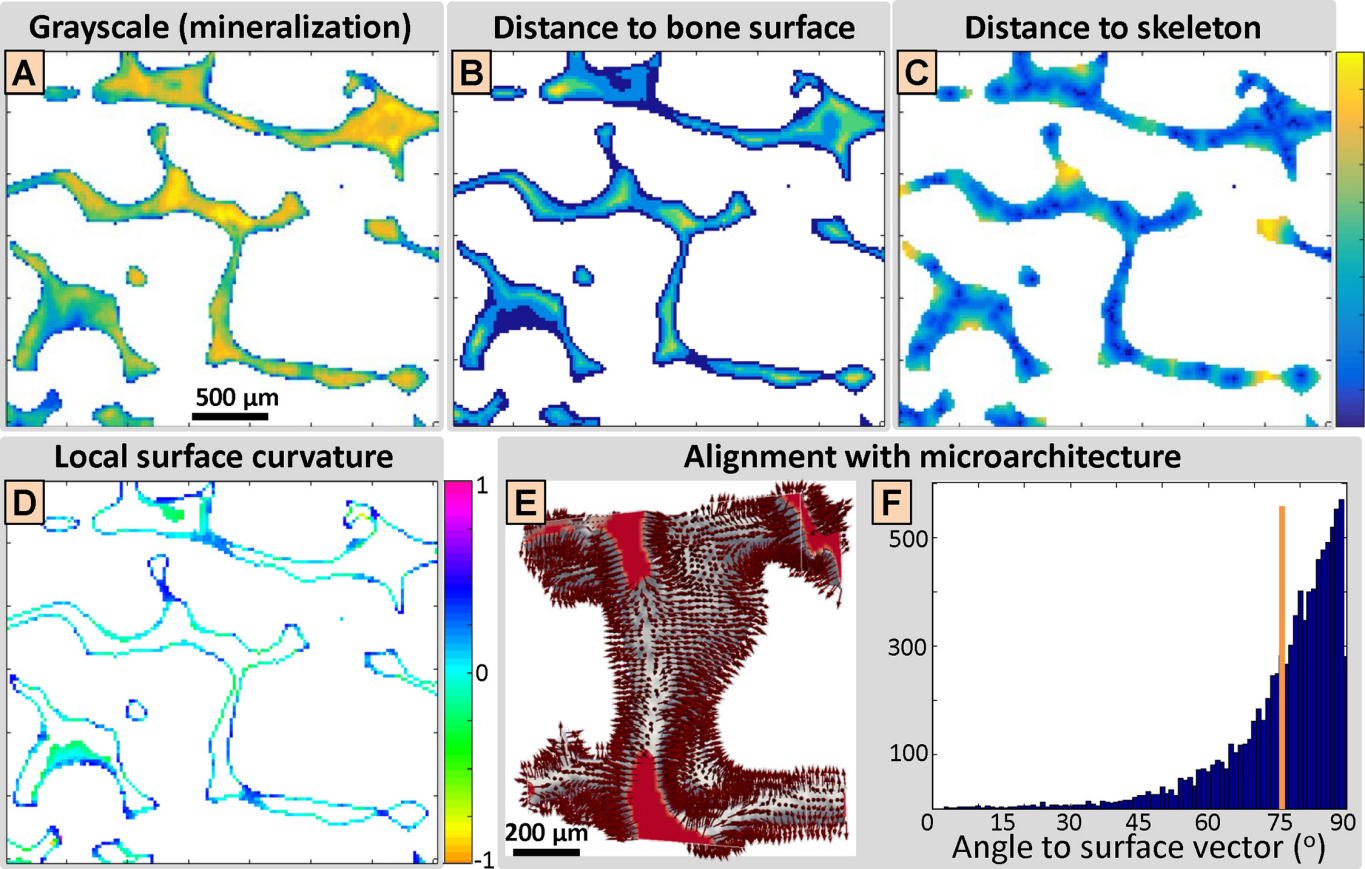
Analysis of local bone ultrastructure in the context of the trabecular microarchitecture. (A) Gray values from synchrotron radiation-based computed tomography (SR CT), which represent local mineralization levels. Linear range: 1–2.5∙10^4^ (a.u.). (B) Distance to bone surface. Linear range: 0–70 μm. (C) Distance to skeleton. Linear range: 0–200 μm. (A-C) Blue (bottom) and yellow (top) represent the minimum and maximal values respectively, where intermediate values are interpreted according to the linear colormap provided at the right border. (D) Local surface curvature. Convex and concave geometries have positive and negative values for the local surface curvature, respectively. (C-D) Distance to skeleton and local surface curvature is heavily affected by the 3D geometry of the trabecular microarchitecture and cannot be easily interpreted in 2D. (E-F) Alignment of the ultrastructure with trabecular microarchitecture. (E) The normals to the bone surface of a trabecula, whose structure and ultrastructure orientation are depicted in Fig 2C and Fig 2D, respectively. (F) The alignment is quantified by the angle of the ultrastructure orientation to the surface normal for each surface element, in the form of a histogram. The orange line visualizes the threshold value of 75° between the two vectors, above which the ultrastructure was considered to be perpendicular to the bone surface and thus aligned with the trabecular microarchitecture.

In order to analyze the local ultrastructure organization in the context of the trabecular microarchitecture, several local measures have been introduced:

Grayscale values (Fig 4A): Grayscale values derived from SR CT scans, which represent local mineralization levels. However, the relationship is not linear, since no calibration phantom has been used.

Distance to bone surface (Fig 4B): Bone surface was defined as object voxels in contact with at least one background voxel, using a 6-connected neighborhood. An absolute grayscale value threshold was selected to segment bone tissue, which was the same for all samples. Distance to bone surface was defined as the distance of each voxel to its closest surface voxel.

Distance to skeleton (Fig 4C): Two different skeletonization procedures with similar outcome were used, based on the medial axis transform using Matlab and on morphological thinning (Image Processing Language or IPL; Scanco Medical AG, Brüttisellen, Switzerland). Distance to skeleton was defined as the distance of each voxel to its closest voxel of the skeleton.

Local surface curvature (Fig 4D): There exist different ways to derive local curvature of a structure, based on fitted spheres at each surface point and its neighboring voxels, on local gradients in *x-*, *y-*, and *z-*direction or on averaged sign changes of normals at the vertices of a triangular surface mesh. Local surface curvature based on averaged sign changes (realized in IPL) provided most relevant representations of the curvature of the trabecular sub-volumes (data not shown here), and was adopted for further analyses.

Alignment of ultrastructure with microarchitecture (Fig 4E and Fig 4F): Ultrastructure alignment was quantified by the angle between the ultrastructure orientation vector derived from 3D sSAXS and the normal vector at each surface point of the trabecular microarchitecture. A threshold value of 75° was chosen, above which the ultrastructure was considered to be almost perpendicular to the surface normal or parallel to the surface and thus aligned with the microarchitecture.

## 3 Results

### 3.1 Validation of 3D sSAXS

**Fig 5:**
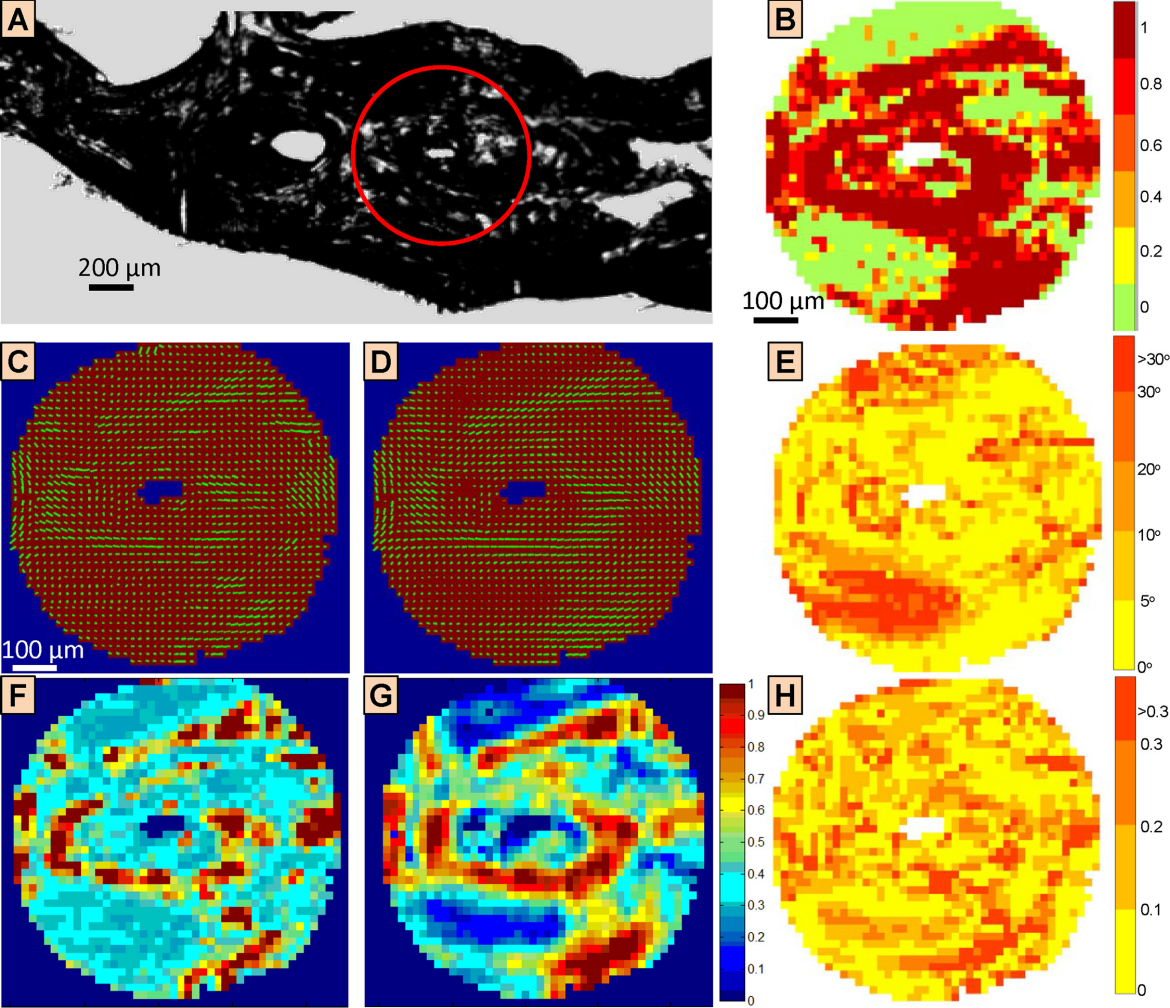
Validation of 3D small-angle X-ray scattering (3D sSAXS) by correlation with linear polarized light microscopy, in terms of ultrastructure orientation and alignment. (A) Bright-field image of bone section, which shows one of the regions of interest (red circle) assessed using both linear polarized light microscopy (PLM) and 3D small-angle X-ray scattering (3D sSAXS). The ROI is circular because of the rotation of the sample for the linear PLM experiments, which leads to a loss of the information at the corners (cf. Fig 3B). (B) The goodness of the sinusoidal fit required in linear PLM to retrieve the ultrastructure orientation, given by the coefficient of determination (R^2^). In most areas, the goodness of the fit was high (> 0.8). However, there were also large areas where the goodness of the fit was very low, which corresponded to areas without any predominant ultrastructure orientation, i.e. very low values of DO. (C) In-plane ultrastructure orientation from linear PLM. (D) In-plane ultrastructure orientation from 3D sSAXS. (C-D) Lines indicate the main orientation of the ultrastructure, where the bar length represents the DO. Local ultrastructure orientations, as obtained from linear PLM and from 3D sSAXS, were comparable. (E) Difference of in-plane ultrastructure orientation between linear PLM and 3D sSAXS. For most points (~75%), in-plane ultrastructure orientation was similar (< 10° difference). Larger differences could be explained by low DO values (Fig 5F and Fig 5G), characterizing areas without any predominant ultrastructure orientation. (F) DO from linear PLM. (G) DO from 3D sSAXS. (H) Difference of DO between linear PLM and 3D sSAXS. (F-H) PLM and 3D sSAXS agreed regarding the location of high and low DO areas. However, high DO areas were bigger when retrieved from 3D sSAXS.

Four ROIs from four different bone sections have been assessed using both linear PLM and 3D sSAXS as described beforehand. Fig 5 depicts one ROI (red circle in Fig 5A) and provides a comparison of the respective linear PLM and 3D sSAXS results (Fig 5B–5H).

The two techniques provided similar results for the in-plane orientation of the bone ultrastructure (Fig 5C and Fig 5D), where the difference was found to be below 10° for most points (~75%) within the ROI (Fig 5E). The choice of the angle from the linear PLM measurements, in terms of the ±90° ambiguity of linear PLM, was confirmed using polarized Raman imaging (see S1 File). However, there are regions where the 2D orientation angle derived from linear PLM and 3D sSAXS differed by more than 30° (~8% of the points). The increased discrepancy in these areas can be explained when examining the coefficient of determination (*R*^*2*^) of the sinusoidal fit of the recorded intensities versus sample orientation from linear PLM (Fig 5B), which provides the (2D) in-plane ultrastructure orientation.

Areas with low degrees of orientation (DOs) as obtained from both techniques (Fig 5F and Fig 5G), which characterize areas where mineralized collagen fibrils were *not* predominantly aligned in one direction, corresponded to a low quality of the fit for linear PLM results (Fig 5B). On the other hand, areas exhibiting high DOs (DO > 0.8), where the ultrastructure was oriented predominantly in one direction, were comparable with areas, where the ultrastructure orientation retrieved from linear PLM and 3D sSAXS was in good agreement (Fig 5E). In other words, where the DO was low and thus, there was no evident predominant orientation of the collagen fibrils observed, a poor quality of the fit of the PLM data to the sinusoidal curve has been encountered, possibly providing in-plane angles that need careful interpretation.

When comparing the DO maps from linear PLM (Fig 5F) and 3D sSAXS (Fig 5G) the locations of both low-DO and high-DO areas were comparable (Fig 5H). However, high-DO areas as obtained from 3D sSAXS (Fig 5G) were more extended and 3D sSAXS seemed to be more sensitive to variations in low-DO areas (DO < 0.5), where PLM discriminated areas of different ultrastructure orientation to a lesser extent (Fig 5F). However, this cannot be attributed to an inherent limitation of PLM as a method, since it depends on the dynamic range of the specific imaging system that is being used. In the range where PLM provided high DO values (DO>0.5), the correlation between the results from PLM and 3D sSAXS was strong (*R*^2^ = 0.85), indicating that both methods are consistent in this range. It should be noted that a thorough analysis of the sensitivity of 3D sSAXS and PLM in terms of the retrieved DOs would require designing, manufacturing and assessing a phantom with known ultrastructure orientation, spanning optimally the range of all possible DOs, which is a non-trivial task, beyond the scope of this work.

### 3.2 3D ultrastructural maps of trabecular bone

Twenty two (22) trabeculae of different alignment, type and size, and from subjects of different ages (Table 1) have been assessed using 3D sSAXS, including a total of 74 histological sections. This involved scanning ~300 ROIs at 10 sample rotation angles each, resulting in ~3000 raster scans and an acquisition of ~7,5 million diffraction patterns. The scanning experiments have been performed during 3 separate beamtime sessions at the cSAXS beamline of the Swiss Light Source in ~33 8-hour shifts and a total of 264.5 hours of 3D sSAXS experiments. Raw data, i.e. the diffraction patterns, amounted to ~18 TB of data. After analyzing these diffraction patterns, extracting the 2D orientation and 2D DO, fitting the data from the different rotation angles to derive the 3D orientation and the 3D DO, registering each ROI to the 3D bone structure and stacking the data from consecutive tissue secitions, the 3D organization maps of the ultrastructure could successfully be derived for all 22 trabeculae. The total disc space required for the resulting analyzed data amounted to ~10 TB.

**Fig 6:**
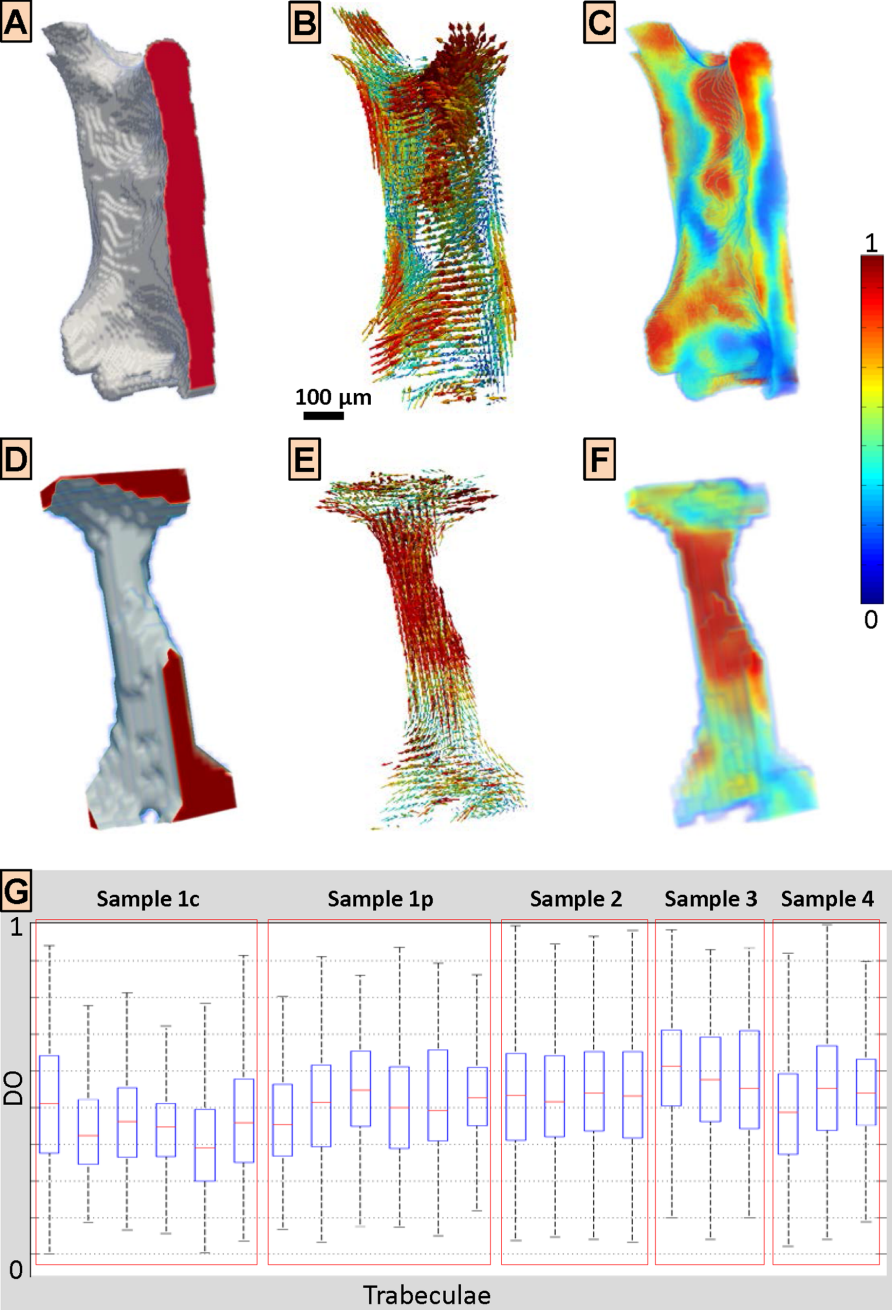
3D scanning small-angle X-ray (3D sSAXS) scattering results for single trabeculae from human vertebral bone. (A-C) Results for trabecula 1 of Sample 1c (cf. Fig 1), which was a plate-type trabecula in craniocaudal direction. (D-F) Results for trabecula 5 of Sample 1p (cf. Fig 1), which was a rod-like trabecula perpendicular to the craniocaudal direction. (A & D) Trabeculae from synchrotron radiation computed tomography at 7.4 μm voxel size. (B & E) Ultrastructure orientation and arrangement represented by vectors. The degree of orientation (DO) is depicted by both the length and the color of the vector, according to the colormap at the right side. Ultrastructure seems to mostly follow microarchitecture. (C & F) DO map, where the colors represent DO values encoded by the colormap at the right side. Distinct domains tens to hundreds of micrometers in size have been identified, representing areas of high DO. (G) Box plot of the observed DO values for all investigated trabeculae, grouped by sample (see Fig 1 and Table 1), indicating median (red line), 25^th^ and 75^th^ percentile (box edges) and lower and upper extreme datapoints. No obvious inter-trabecular and intra-trabecular differences have been observed for the DO values.

Ultrastructure organization obtained from 3D sSAXS, mapped on trabecular structure from SR CT, is shown in Fig 6A–6F for two representative trabeculae; a plate-like trabecula in craniocaudal orientation and a rod-like trabecula from a perpendicular anatomical direction. For both cases, the ultrastructure followed the trabecular microarchitecture (Fig 6B and Fig 6E). More specifically, the ultrastructure seemed to be organized in domains of tens to hundreds of micrometers in size representing areas of high DO, where the high-DO elements were located mostly closer to the bone surface with areas of low DO in-between (Fig 6C and Fig 6F). Moreover, a closer analysis of the DOs, grouped by sample (Fig 6G), shows that no obvious differences for inter-trabecular DOs and DO variations have been identified.

### 3.3 Analysis of bone ultrastructure and trabecular microarchitecture

Each 20 × 20 × 20 μm^3^ volume of the investigated trabecular bone structure within this first 3D sSAXS study was characterized by the orientation and the DO of its ultrastructure. Moreover, we introduced and analyzed additional measures to quantify ultrastructure organization in the context of the trabecular microarchitecture, such as *distance to bone surface*, *distance to the skeleton*, *local surface curvature* or *alignment with microarchitecture* as defined in the Materials and methods section. The analysis of bone ultrastructure in the context of the trabecular microarchitecture is presented here.

#### 3.3.1 Bone ultrastructure follows trabecular microarchitecture

**Fig 7:**
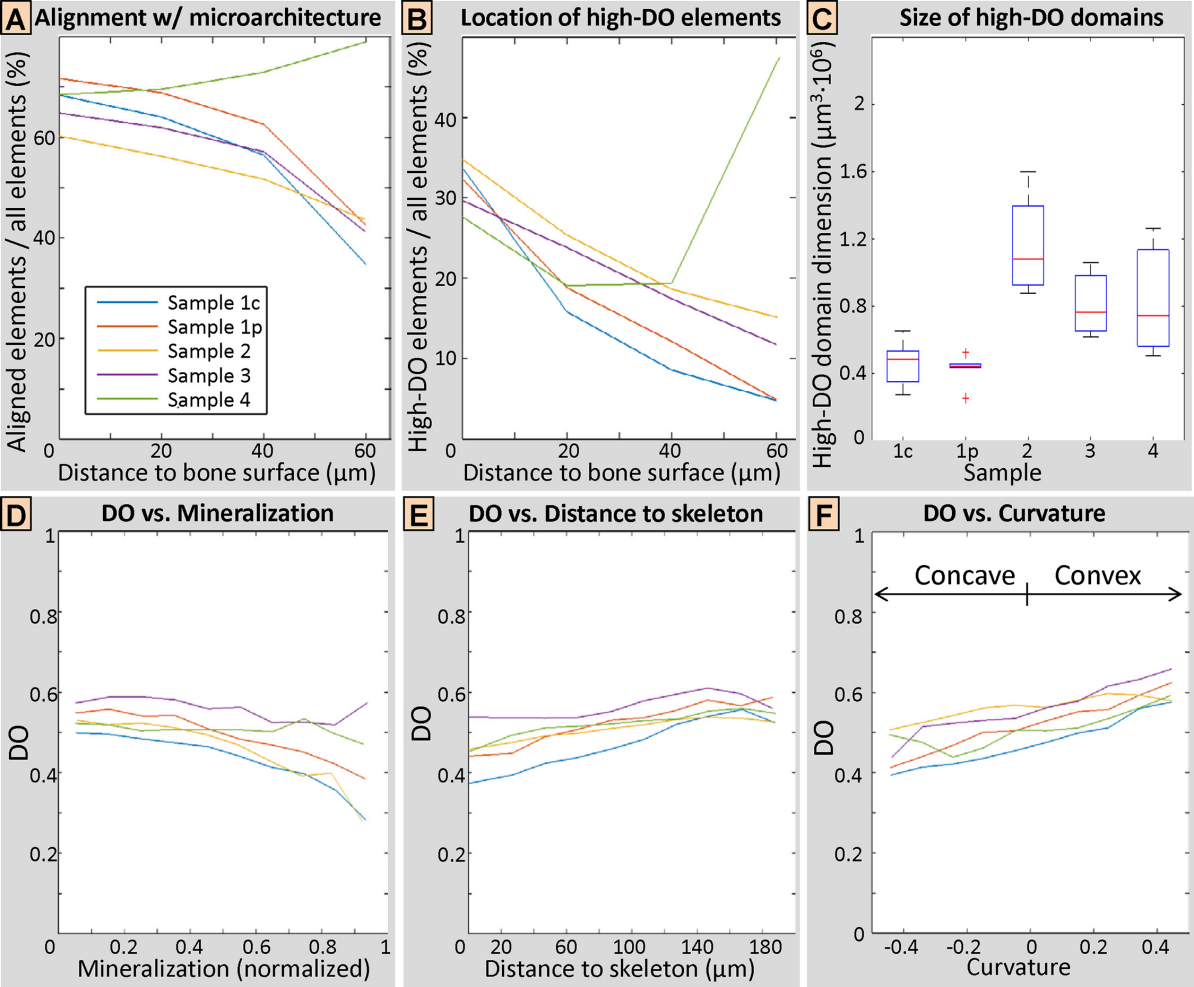
Bone ultrastructure and trabecular microarchitecture. (A) Elements of the ultrastructure aligned with the microarchitecture, plotted against the distance to bone surface. At the bone surface (distance to bone surface = 0), the majority of the ultrastructure (~60–75%) was aligned with the trabecular microarchitecture. Towards the trabecular core, at 50 μm from the bone surface, this percentage dropped to about ~50%. A random ultrastructural organization would have resulted in a percentage of ~17% only. Sample 4 (from the 77 y.o. male donor) showed an opposite behavior, where the ultrastructure towards the trabecular core was more prominently oriented along the bone surface than the ultrastructure close to the surface. (B) Plot of the percentage of ultrastructural elements with high degree of orientation (DO) against the distance to bone surface. Highly oriented ultrastructural areas were preferentially found closer to the bone surface and constituted about 30% of the bone tissue close to the trabecular surface. Sample 4 (from the 77 y.o. male donor) showed the opposite behavior, where ultrastructural areas with highest DOs were found preferentially towards the trabecular core. (C) Box plot of high-DO domain dimensions, indicating median (red line), 25th and 75th percentile (box edges) and lower and upper extreme datapoints. Ultrastructural domain dimensions were in the order of tens to hundreds of μm. (D) Plot of DO against grayscale values, indicating local mineralization levels. Areas with higher DO were mostly inversely related to local mineralization levels. (E) Plot of DO against distance to skeleton. DO was mostly positively related to the distance to the skeleton. (F) Plot of DO against local surface curvature. DO was mostly positively related to local surface curvature.

At the surface: The first plot in Fig 7A, which relates trabecular microstructure and local ultrastructure in terms of the *alignment of ultrastructure with microarchitecture* plotted against distance from bone surface, confirmed that for all samples, the ultrastructure close to the surface was primarily aligned with the actual trabecular surface. In numbers, the ultrastructure of the surface elements (distance to surface = 0) was in most cases (between 60% and 72%) aligned with the bone surface, where the lowest percentage was observed for Sample 2 from the 27 y.o. male subject. It should be noted that a random ultrastructure organization would have resulted in a percentage of ~17% only.

Inside the trabecula: In 4 out of the 5 samples the effect of the alignment with microarchitecture is alleviated when going towards the core of the trabecula. This indicates that the relation between bone surface geometry and ultrastructure was less pronounced, but still around 50% of the ultrastructure was aligned with the bone surface at a distance of ~50 μm from the bone surface. One exception is the sample from subject 4 (77 y.o. male), where the ultrastructure towards the trabecular core was more prominently oriented with the microarchitecture than the ultrastructure in proximity to the surface.

In addition, we investigated how different structural or ultrastructural attributes such as the local curvature or the DO can affect the alignment of the ultrastructure to the microarchitecture and concluded that they do not play an important role (data not shown here).

#### 3.3.2. Highly oriented ultrastructural areas are located closer to bone surface

The data in Fig 7B represent the percentage of voxels showing high DO values (defined as the voxels above the 75% DO percentile, with DO > 0.64) over all elements within the bone tissue, plotted against the *distance to bone surface*. Fig 7B shows that highly orientated ultrastructural areas were mostly located closer to the bone surface and made up about 30% of the bone tissue in the proximity of the trabecular surface, and they were less abundant towards the core of the trabecula. This trend was reversed for subject 4 (77 y.o. male), where ultrastructural domains exhibiting highest DOs were found preferentially towards the trabecular core.

#### 3.3.3 Ultrastructure is arranged in domains

The data in Fig 7C show that the dimensions of high-DO domains (DO > 0.64) are in the range of tens to hundreds of micrometers in each dimension. The mean cluster size of ~0.75·10^6^ μm^3^ would correspond to a to a cubic ~90×90×90 μm^3^ domain. Since the high-DO elements were preferentially located close to the bone surface, ultrastructural domains were rather elongated with typical dimensions of ~40×135×135 μm^3^ for instance. When investigating the lower DO elements, it was not possible to divide them in discrete domains, since they formed a rather extended and connected compartment. This indicates that the high-DO domains were separated by lower DO areas, which made most of the (trabecular) bone tissue (see also Fig 7B).

#### 3.3.4 Ultrastructural degree of orientation and trabecular microarchitecture

Grayscale values: DO was mostly inversely related to grayscale values from SR CT and hence, to local mineralization levels (Fig 7D). In other words, bone tissue with higher DO values was more likely to yield a lower mineralization level.

Distance to skeleton: DO was mostly positively related to distance to skeleton (Fig 7E). In other words, elements closer to the core of the trabecula had typically lower DO values. However, compared to the *distance to bone surface* (Fig 7B), the relation with DO seemed to be weaker, while Sample 4 from the oldest subject in the current study (77 y.o. male) did not show any exceptional behavior.

Local surface curvature: DO was mostly positively related to local surface curvature (Fig 7F). This means that the more concave the bone surface (negative curvature), the lower the DO, and the more convex the surface (positive curvature), the higher the DO.

## 4 Discussion

### 4.1 Validation of 3D sSAXS

Polarized light microscopy (PLM) has been the most commonly used technique to study bone ultrastructure organization. Linear PLM provides the (2D) in-plane orientation of the bone ultrastructure by rotating either the sample or the laser polarization and by fitting the detected intensity to a sinusoidal curve [12], although there is always a ±90° ambiguity concerning the actual orientation [11] (see S1 File). 3D sSAXS is a recently developed technique to quantitatively derive the 3D orientation and the degree of orientation (DO) of the mineralized collagen fibrils [29]. To validate 3D sSAXS using linear PLM, we compared the information obtained from linear PLM and 3D sSAXS regarding ultrastructure orientation and arrangement of 20-μm-thick bone tissue sections. Comparisons between 2D results from PLM and SAXS have been explored in the past [33,43,44]. However they were always limited to qualitative assessments, based on visual observations. Here, we have projected the 3D sSAXS orientation data from 3D to 2D, with the aim to quantitatively validate 3D sSAXS results using linear PLM. Linear PLM has been chosen as gold standard in this study.

The quantitative comparison showed that the ultrastructural results provided by 3D sSAXS are very similar to those retrieved from linear PLM experiments, both in terms of in-plane ultrastructure orientation and in terms of DO. Discrepancies in the in-plane orientation between the outcomes from the two techniques have been identified in bone volumes where the DO was low. Generally speaking, where the DO was low and hence, there was not an evident predominant orientation of the collagen fibrils observed, the in-plane angles from both techniques need careful interpretation. The same observation has been made previously for the fit of 3D sSAXS data [29], which is required to determine the 3D ultrastructure orientation.

An important limitation of the comparison between 3D sSAXS and linear PLM is due to the ±90° ambiguity involved when deriving the in-plane fibril orientation using linear PLM. This limitation has been overcome by choosing the in-plane fibril orientation from linear PLM that was closer to the projected 3D sSAXS data, which was verified *post hoc* using polarized Raman imaging (PRI) (see S1 File). It should be noted that PRI requires more expensive equipment than PLM and is much slower, where acquisition of a spectrum corresponding to a single point in PRI typically requires tens of seconds, compared to milliseconds needed to acquire a whole 2D image in PLM. In addition, proper quantification of band peaks in PRI needs appropriate algorithms and knowledge of the chemical bonds of the tissue as well as of the embedding material.

### 4.2 3D ultrastructural maps of trabecular bone

The 3D ultrastructure organization of completebone trabeculae and its quantitative analysis is presented here for the first time, by providing 3D ultrastructural maps of trabecular bone using 3D sSAXS. Previous studies on bone ultrastructure orientation and arrangement were possible either for very small volumes of interest by using 3D electron microscopy [27,45], ptychography [24,25] or phase nano-tomography [21–23] (volume edges in the order of tens of micrometers), or were restricted to surface/2D analyses using scanning electron microscope (SEM) [46], sSAXS [33] and/or PLM [43]. A detailed description and comparison of the methods used for studying organization of bone ultrastructure, including recent developments, is presented in [28]. It should be noted that although 3D sSAXS was used to quantify ultrastructure organization in trabecular bone, the method can also be used in the analysis of mineralized collagen fibril orientation and arrangement of cortical bone without changes to the experimental setup or the data processing and analysis methods presented here.

Visual inspection of the derived ultrastructure organization allows first qualitative observations, such as the fact that the trabecular ultrastructure seems to be aligned with the tissue architecture of the respective trabecula, which agrees for instance with qualitative observations of trabecular bone surfaces based on SEM [46]. Furthermore, areas of bone ultrastructure exhibiting a higher DO appeared to be located preferentially closer to the trabecular bone surface. This agrees with visual observations of trabecular ultrastructure in 2D [27,47]. Highly oriented ultrastructural areas also seemed to be usually arranged in domains with dimensions in the order of tens to hundreds of μm, separated by trabecular bone of lower DO. This finding resembles patterns that have been reported for various length scales in bone tissue, either for trabecular bone, where oriented ultrastructural motifs are surrounded by randomly arranged areas at the sub-micrometer scale [27], or for cortical bone, exhibiting oriented and randomly arranged sub-micrometer layers or domains [48,49] and showing ordered and disorder motifs at a micrometer scale [26]. Reconstruction of the ultrastructure organization of 22 trabeculae, involving assessment of 74 individual 20-μm-thick bone sections, required 164.5 hours of SAXS experiments, excluding data processing and evaluation. This fact demonstrates that the current method is not yet close to a level where it can be considered for clinical use. However, very recently, Kagias and co-workers demonstrated the possibility of retrieving 2D ultrastructure orientation by exploiting the ultra-small-angle X-ray scattering signal of grating interferometry in a single shot over millimeter-sized fields of view [50]. Further, Liebi and co-workers developed a method that extends the concept of X-ray tomography from scalar (tissue density) values to tensors, by enabling non-destructive tomographic reconstruction of bone ultrastructure organization using X-ray scattering information [19]. These developments for the assessment of bone ultrastructure organization bring SAXS technologies closer to applications in a pre-clinical and clinical context.

### 4.3 Structure-ultrastructure correlations

Quantitative analysis of the 3D sSAXS and SR CT datasets provided different relationships between the orientation and the DO of the ultrastructure and the trabecular microarchitecture. First, ultrastructure orientation was found to follow the trabecular microarchitecture, most prominently in the vicinity of the trabecular surface and more weakly towards the core of the trabecula. A correlation of the direction of the ultrastructure organization and the trabecular microarchitecture has been suggested in previous studies based on observations from thin sections [33] or the trabecular surface [46], but here, to our best knowledge, it is for the first time that such a relation has been quantitatively investigated in 3D and in relation to the distance to the trabecular surface. An exception was the sample from the oldest subject in the current study (77 y.o. male), where the bone tissue close to the trabecular core was aligned to a greater extent than towards the bone surface. Possibly, this is result of the changed bone metabolism in old and/or osteoporotic individuals, where bone tissue at the surface is continuously being resorbed and the surface geometry altered accordingly. It is possible that the ultrastructure not aligned with the trabecular microarchitecture is preferentially resorbed with age, and at the same time, the ultrastructure that is already aligned is preserved. This would be similar to the effect of aging and/or osteoporosis on the microarchitecture of vertebrae, where trabeculae become gradually more intensely aligned with the craniocaudal/main loading direction [51–53]. Despite the fact that this reverse trend was observed for all three trabeculae of the elder subject, in contrast to all trabeculae of the other subjects, experiments including more subjects are needed to investigate such a hypothesis in more detail. It should be noted that the quantification in terms of distance to the trabecular surface has been conducted in increments of 20 μm, corresponding to the edge of the probed cubic volumes. Use of thinner sections and smaller X-ray beam and motor step sizes will result in smaller probed cubic volumes and can thus enable higher-resolved quantification, albeit at the expense of volume of interest that can be analyzed in the same amount of time.

Furthermore, areas of high DO were found preferentially close to the trabecular surface, and comprised ~30% of the bone tissue in proximity of the surface. The decreasing percentage of highly oriented ultrastructure towards the trabecular core might be connected to differences in trabecular function, since outer parts of trabeculae might experience higher tensile strains, and thus need to have an appropriate and more dominant fibril orientation [54,55]. Interestingly, the sample from the oldest subject in the current study (77 y.o. male) showed the opposite behavior, where ultrastructural domains with highest DOs were found preferentially towards the trabecular core instead.

Concerning the size of the high-DO domains, they were found to be in the range of 0.75∙10^6^ μm^3^, which would correspond to a domain size of ~50×130×130 μm^3^ for instance. The suggested 50 μm for the depth of the domain lies within the interval of 40–60 μm from the distance to the surface in Fig 7A, where beyond this distance and towards the trabecular core, the plot representing the elements of the ultrastructure aligned with the microarchitecture changes its behavior considerably. The observed domain size is similar to the size range found for bone remodeling sites [56], ~50×20–50×100 μm^3^. Since the depth of a typical bone remodeling site is also ~50 μm, one suggestion is that high DO areas are laid down during a single remodeling activity of a bone remodeling unit. Moreover, to our best knowledge, bone surface curvature has been correlated here for the first time to the organization of the bone ultrastructure. Namely, the relation of the DO with the local surface curvature was positive in all cases; concave sites tended to have a lower DO, whereas convex areas had an ultrastructure that was more prominently oriented in one direction. Bone surface curvature has been correlated to tissue growth [57]: the more concave the surface, the higher the tissue growth [58], while higher surface convexity leads to lower tissue growth [59].

## 5 Conclusions

In the current study, we successfully validated 3D sSAXS using linear PLM, regarding assessment of ultrastructural orientation and arrangement. In addition, we presented the first 3D sSAXS study providing 3D ultrastructure organization maps of trabecular bone, for 22 trabeculae of different alignment, types and sizes, and from subjects of different ages. The presented 3D ultrastructural maps of bone tissue represent an important step towards understanding how the mineralized collagen fibrils are organized, in the context of the bone microarchitecture.

## Supporting Information

S1 File PLM angle ambiguity & polarized Raman imaging. The angle ambiguity associated with linear PLM is mathematically explained, the reason why this ambiguity is not present in polarized Raman imaging is demonstrated, and the use of polarized Raman imaging to overcome the ambiguity in linear PLM in this study is presented.
(DOCX)

S2 File 3D sSAXS information for all 22 trabeculae. The 3D sSAXS information is provided for all 22 trabeculae, as these are presented in Table 1. For each trabecula, the X, Y and Z component of the vector that represents the ultrastructure organization at any given point of the structure is given. For example, for the 3^rd^ trabecula of Subject 2, the variable that contains its ultrastructure organization is “Trab2_3”. In the 4-dimensional variable, the first dimension corresponds to the X-, Y- and Z-component of the vectors, and the other three form the 3-dimensional matrix of the trabecular structure. As an example, the Z-component is stored as (3, : , : , : ).
(MAT)
