# Supplementary material for: Ultrastructure Organization of Human Trabeculae Assessed by 3D sSAXS and Relation to Bone Microarchitecture
Source: PLoS One. 2016 Aug 22;11(8):e0159838. doi: 10.1371/journal.pone.0159838 (PMC4993496; doi:10.1371/journal.pone.0159838)
Supplement: S1 File — The angle ambiguity associated with linear PLM is mathematically explained, the reason why this ambiguity is not present in polarized Raman imaging is demonstrated, and the use of polarized Raman imaging to overcome the ambiguity in linear PLM in this study is presented. (DOCX) [file pone.0159838.s001.docx]

# PLM angle ambiguity & polarized Raman imaging

There is an inherent ambiguity of ±90^o^ to derive ultrastructure orientation using linear PLM. This is because of the use of crossed-polarizers on the microscope setup.. In the experimental setup, we consider three elements in series, which the light has to go through: i) the microscope (linear) polarizer, which is at an angle that we define as 0^o^, ii) the birefringent mineralized collagen fibrils that act as polarizers, at a random angle *α*, and iii) the analyzer (or second linear polarizer) of the setup, at an angle of 90^o^_­_ to the first polarizer and an angle $90-\alpha$ to the fibrils. Given that the first polarizer results in a linearly-polarized beam, and that the transmittance of an inclined polarizer is given ([1](#_ENREF_1)) by:

$T=\left( T_{1}-T_{2} \right)\cos^{2} \alpha+T_{2}$, Eq. (S1)

where $T_{1}$and $T_{2}$are the so-called principal transmittances, i.e., the maximum and the minimum values of the transmittance, respectively, with T_1_$\gg$T_2_,

then the collective transmittance of the collagen fibrils (c) and the analyzer (a) is:

$T_{tot}=T_{c}T_{a}\cong\left( T_{1c}-T_{2c} \right)\left( T_{1a}-T_{2a} \right)\cos^{2} \alpha\sin^{2} \alpha$. Eq. (S2)

$T_{tot}$ has a period of 90^o^, is maximal at $a={45}^{o}+n{90}^{o}$ and minimal at $a=n{90}^{o}$, where *n* is an integer. An example for *n* = 0 is shown in Fig. 3.

For validation of the 3D sSAXS results regarding bone ultrastructure orientation, through correlation with 2D PLM data, the inherent ±90^o^ ambiguity of PLM in terms of ultrastructure orientation had to be addressed. In this study, this limitation has been overcome by selecting the PLM-derived in-plane orientation, which was closer to the 3D sSAXS outcome. This choice needed verification, which was performed using polarized Raman imaging (PRI).

Compared to PLM and SAXS, PRI is a more recent imaging modality, which can be used to investigate bone ultrastructure organization ([2](#_ENREF_2)). The direction of the collagen fibrils affects specific bands in the Raman spectrum, such as the amide-I and the *v*_1_ phosphate band ([3](#_ENREF_3)), which allows examining the orientation of the mineralized collagen fibrils. The areas under the peaks of these bands can be analyzed similarly to the intensities observed in PLM (Fig. A in S1 file), since they both follow a sinusoidal curve when plotted against the rotation angle of the sample ([4](#_ENREF_4)). However, due to the lack of an analyzer in the imaging setup, the governing equation is reduced to Eq. (3), which has a period of 180^o^. Consequently, there is no ±90^o^ ambiguity for PRI known from linear PLM, and the in-plane ultrastructure orientation can be retrieved unambiguously.

Seven randomly selected points of a single histological section from Sample 2 that was also examined with PLM and 3D sSAXS have been investigated with PRI, to verify the choice of the ultrastructure orientation retrieved from PLM and based on a comparison with 3D SAXS data. The PRI experiments have been performed on a confocal Raman spectroscope (CRM200; WITec, Germany) with a diode-pumped green, linearly polarized laser excitation (λ = 532 nm, beam diameter (FWHM) = 2 μm; WITec, Germany) and a 20X microscope objective (NA=0.4; Nikon, Japan). The direction of the laser polarization has been rotated in 7 steps from 0° to 180° with a λ/2 plate. Spectra were acquired using a cooled CCD camera (WITec, Germany) behind a grating spectrograph (900 grooves/mm). An integration time of 20 s, a 6× hardware accumulation and a 1× software accumulation have been applied.

The areas under the Raman peaks have been extracted using an in-house Matlab algorithm. In order to maximize the angle-dependency effect of the areas under the peak in terms of the sample rotation, we used the ratio of the areas under the two inversely affected peaks - the amide-I and the *v*_1_ phosphate peak. These peaks were also found to be the most sensitive to changes in sample orientation (see Fig. A in S1 file). The ratio between these two peaks is also referred to as “mineral-to-matrix ratio” ([5](#_ENREF_5)). The sinusoidal fit was performed in Matlab.


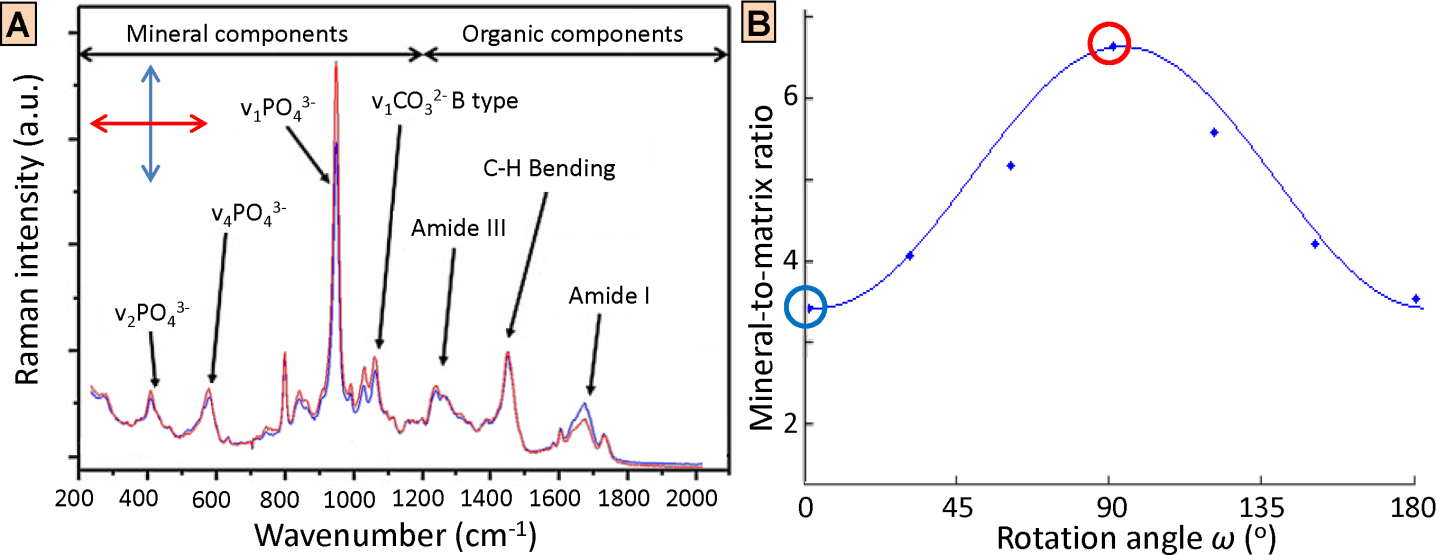


**Fig A. Procedure to retrieve ultrastructure orientation using polarized Raman imaging.**

(A) Two experimental polarized Raman spectra of bone tissue with most important mineral and organic peaks labelled, acquired under orthogonal laser polarization directions (red and blue double-headed arrow inset). The peaks, which are mostly affected by the change in laser polarization direction, are the v_1_ phosphate peak (~960/cm^-1^) and the amide-I peak (~1650/cm^-1^), as shown by the different areas under the peaks. Peak assignments according to data in ([6](#_ENREF_6)). (B) Plot of the peak height ratio between the v_1_ phosphate and the amide-I band, which is a measure of the mineral-to-matrix ratio or tissue mineralization ([5](#_ENREF_5)), plotted against sample rotation angle, which allows retrieving the in-plane orientation of the mineralized collagen fibrils. The red and blue circles indicate the orthogonal laser polarization direction as shown in (A).

# References

1. Bennett JM. Polarization. In: Bass M, editor. Handbook of optics. 1. 2 ed: McGraw - Hill; 1995.

2. Kazanci M, Wagner HD, Manjubala NI, Gupta HS, Paschalis E, Roschger P, et al. Raman imaging of two orthogonal planes within cortical bone. Bone. 2007;41(3):456-61.

3. Galvis L, Dunlop JWC, Duda G, Fratzl P, Masic A. Polarized Raman anisotropic response of collagen in tendon: Towards 3D orientation mapping of collagen in tissues. PLoS One. 2013;8: e63518(5).

4. Rousseau ME, Lefevre T, Beaulieu L, Asakura T, Pezolet M. Study of protein conformation and orientation in silkworm and spider silk fibers using Raman microspectroscopy. Biomacromolecules. 2004;5(6):2247-57.

5. Morris MD. Raman spectroscopy of bone and cartilage. In: Matousek P, Morris MD, editors. Emerging Raman Applications and Techniques in Biomedical and Pharmaceutical Fields. Biological and Medical Physics Biomedical Engineering: Springer, 233 Spring Street, New York, Ny 10013, United States; 2010. p. 347-64.

6. Kozielski M, Buchwald T, Szybowicz M, Blaszczak Z, Piotrowski A, Ciesielczyk B. Determination of composition and structure of spongy bone tissue in human head of femur by Raman spectral mapping. J Mater Sci Mater Med. 2011;22(7):1653-61.
